# Advances in Regenerative Medicine for Orthopedic Injuries: A Comprehensive Review

**DOI:** 10.7759/cureus.79860

**Published:** 2025-02-28

**Authors:** Samyabrata Das, Amit Thakur, Anupam Datta, Ayaskant Sahoo, Sukanta Bandyopadhyay, Ashok K Sah

**Affiliations:** 1 Orthopaedic Surgery, Sri Ramachandra Institute of Higher Education and Research, Chennai, IND; 2 Department of Orthopaedics and Traumatology, All India Institute of Medical Science Jammu, Jammu, IND; 3 Forensic Medicine, Agartala Government Medical College &amp; Govind Ballabh Pant (GBP) Hospital, Agartala, IND; 4 Anaesthesia, NRI Institute of Medical Sciences, Visakhapatnam, IND; 5 Biochemistry, Rama Medical College Hospital Research Centre, Kolkata, IND; 6 Department of Medical Laboratory Sciences, College of Applied and Health Sciences, A Sharqiyah University, Ibra, OMN

**Keywords:** 3d bioprinting, growth factors, mesenchymal stem cells, orthopedic applications, regenerative medicine, tissue engineering

## Abstract

Orthopedics is one field that greatly benefits from the new ideas provided by regenerative medicine. This review pulls together the most recent publications involving stem cell therapy, platelet-rich plasma, growth factor, gene therapy, tissue engineering, stem cell-derived extracellular vesicles, and other regenerative technologies in the context of bone, cartilage, tendon, and ligament healing. Recent studies show that these new therapies can alter cell development, division, and production of fiber and ground substance to remodel tissues. Nevertheless, the clinical application has several issues such as the standardization of cell procurement and preparation, the control of cytokine/gene delivery, the revascularization of tissues, and the requirements of large samples, positively controlled clinical trials. More research must be conducted to overcome such barriers and make practicing more applicable in real life.

## Introduction and background

Orthopedic conditions, which involve bone, cartilage, tendon, and ligament, are extremely common; millions of people all over the world are affected by them annually [1]. These injuries may be extremely disabling and, in most instances, fail to respond well to therapy and surgical procedures [2]. Therefore, there has been a growing focus on employing regenerative medicine methodologies to facilitate tissue repair and regeneration in orthopedic tissues [3, 4]. Regenerative medicine takes advantage of the human body's healing mechanisms to repair cells and tissues that have been damaged [5]. This review summarizes the uses of regenerative medicine in addressing many of the usual orthopedic traumas.

Orthopedic injuries include fractures, ligament or tendon tears, articular cartilage trauma, and intervertebral disc injuries [1]. It is not unusual to sustain fracture, and injury to soft connective tissues in joints is experienced frequently, especially in the physically working population [2,6]. They also found that cartilage damage can also occur in this lesion, and chondrocyte degeneration leads to joint pain and osteoarthritis in the long term [7]. Other spinal disorders, such as disc degeneration, are also issues [8]. These various orthopedic injuries, as have been indicated, affect the quality of life, and most of them do not heal with ordinary treatment [3]. As a result, regenerative techniques seek to enhance the ability of tissues to heal themselves naturally.

Autologous treatments such as stem cell treatment, platelet-rich plasma, and tissue engineering involve the use of substances in the body or biomaterials to promote regeneration [5,9,10]. Bone marrow, adipose tissue, or synovial fluid resident MSCs have the potential to form bone, cartilage, fat, and other lineages [5]. For this reason, platelet-rich plasma (PRP) contains growth factors that promote healing as well [9]. Scaffolds and hydrogels can also promote regeneration of tissues [10]. These treatments work with the healing ability of the body.

**Figure 1:**
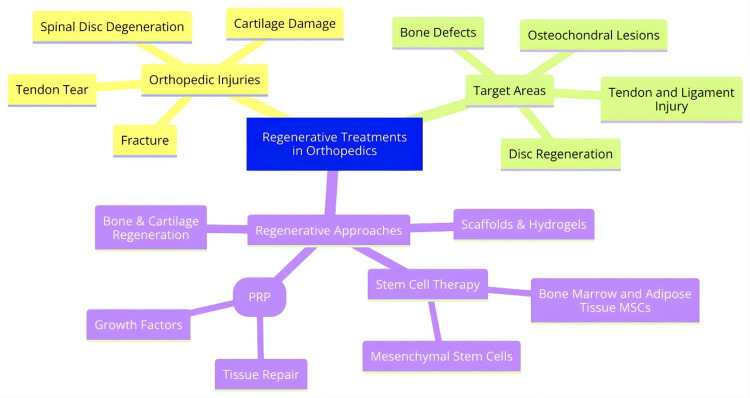
Regenerative treatments in orthopedics The image was created by the authors of this article.

The use of regenerative medicine in orthopedics is as follows: In the case of bone defects, it has been shown that the use of mesenchymal stem cells on biomaterial scaffolds holds potential for bone regeneration [11]. In the treatment of osteochondral lesions, are stem cell delivery with scaffolds useful both for bone and cartilage repair [12]. PRP and stem cells also cure tendon and ligament damage as well [13,14]. For spinal disorders, treatments to halt disc degeneration or even regenerated nucleus pulposus cells are also being investigated [15]. Thus, regenerative injectables are non-surgical or minimally invasive cosmetic procedures. Tissue-engineered constructs can, in addition, reinstate body structures that have been destroyed in Figure 1. In general, various regenerative treatments for typical orthopedic complex injuries are beginning to be developed.

Orthopedic injuries that are unresponsive to ordinary treatment are often found in practice. A new field like stem cell and PRP therapy and tissue engineering, however, uses the body's ability to self-heal and has the potential to further enhance the result of various types of orthopedic problems. Such research in this area is to explore additional reactivation solutions for conveying numerous regenerative treatments into larger clinical practice.

## Review

Principles of regenerative medicine in orthopedics

Healing of wounds and restoration of tissue are multifaceted biochemical events occurring at both the species and molecular levels. Several critical processes are evidenced to participate in the tissue repair event, such as inflammation, cell proliferation, angiogenesis, fibrosis as well as deposition of extracellular matrix (ECM), stem cell differentiation to form specific lineages including osteogenic, chondrogenic differentiation, and the more recent concepts of vasculogenic. All these mechanisms on their own or in parts should be in balance for the proper process of regeneration to happen in Table 1.

**Table 1:** Key concepts and biological mechanisms of tissue repair, as well as cellular and molecular biology aspects ECM: Extracellular matrix; VEGF: Vascular endothelial growth factor; PDGF: Platelet-derived growth factor; TGF-β: Transforming growth factor-beta; MMPs: Matrix metalloproteinases; TIMPs: Tissue inhibitors of metalloproteinases; BMPs: Bone morphogenetic proteins

Concept	Mechanism	Cell Type Involved	Molecular Signals	Role in Tissue Repair	Challenges
Inflammation	Initial immune response to injury	Macrophages, neutrophils	Cytokines (IL-1, TNF-α)	Clears debris, promotes repair	Excessive inflammation causing damage
Cell Proliferation	Cell division for tissue regeneration	Fibroblasts, endothelial cells	Growth factors (VEGF, PDGF)	Rebuilds tissue structure	Uncontrolled proliferation (tumor risk)
Angiogenesis	Formation of new blood vessels	Endothelial cells	VEGF, FGF	Supplies oxygen and nutrients	Abnormal vessel growth
Fibrosis	Scar tissue formation	Fibroblasts	TGF-β, collagen synthesis	Provides temporary matrix for repair	Excessive fibrosis, tissue stiffness
ECM Remodeling	Reorganization of extracellular matrix	Fibroblasts, matrix metalloproteinases (MMPs)	MMPs, TIMPs	Restores tissue architecture	Imbalanced ECM production
Stem Cell Differentiation	Stem cells become specialized cells	Mesenchymal stem cells (MSCs)	Wnt, Notch signaling	Regenerates damaged tissues	Low efficiency, cell sourcing issues
Osteogenesis	Bone formation	Osteoblasts, osteoclasts	BMPs, TGF-β	Bone regeneration	Delayed healing in complex fractures
Chondrogenesis	Cartilage formation	Chondrocytes	SOX9, TGF-β	Cartilage repair	Limited cartilage regeneration
Vascularization	Formation of new capillaries	Endothelial cells, pericytes	VEGF, angiopoietin	Ensures nutrient delivery	Uncontrolled growth (vascular diseases)
Apoptosis	Programmed cell death	Various cell types	Caspases, Bcl-2	Removes damaged cells, prevents cancer	Excessive apoptosis leads to tissue loss

Essentially, during the inflammation phase, immune system cells such as macrophages and neutrophils indicate that there is a problem and also help to remove debris from the injured area. It is triggered by cytokines release such as interleukin-1 (IL-1) and tumor necrosis factor-alpha (TNF-α). After that, cell proliferation reconstructs the loss of tissue by stimulating the growth factor of fibroblast, endothelial, and other cells such as vascular endothelial growth factor (VEGF) and platelet-derived growth factor (PDGF). Angiogenesis dilutes and replaces areas of low circulation using VEGF, fibroblast growth factor (FGF), and other signals. The initial stabilization during fibrosis is provided by the ECM, which is temporarily developed with collagen through fibroblasts stimulated by TGF-β. Thus, as the healing advances, matrix metalloproteinases (MMPs), and tissue inhibitors of metalloproteinases (TIMPs) reconstruct fibrosis into more and more physiological matrix. According to Wnt, Notch, other factors of stem cell differentiation help to rebuild damaged kinds of cells. These other more recently investigated pathways are osteogenesis (formation of bones through BMPs and TGF-β) [16], chondrogenesis (cartilage formation via SOX9 and TGF-β), and vascularization signals such as VEGF and Angiopoietin that is supportive of vessel development. Finally, but still of great importance, apoptosis removes cells that have been harmed facilitated by caspases and Bcl-2 proteins [17].

Stem cell therapies for orthopedic injuries

There are several fundamental biological reaction systems that tissue repair and regeneration contain, such as inflammation, cell proliferation, angiogenesis, cell-matrix remodeling, and fibrosis. The inflammatory phase begins very soon after an injury, when white blood cells, specifically macrophages and neutrophils, move to the wound site and release cytokines, which include IL-1 and TNF-α, to clear debris. Cell proliferation consists of the growth necessary to replace lost tissue and is performed by fibroblasts, vasodilator endothelial cells, and other differentiated cell types in reaction to cytokines such as VEGF and PDGF. Healing tissue is supported with freshly formed networks of blood vessels from existing vasculature through endothelial cells that depend on gradients of factors such as VEGF and FGF that instruct cells to form new vessels. Fibroblasts deposit scar tissue through the secretion of ECM and collagen through the activation of TGF-β, hence offering a provisional environment for scaffold structure. Last of all, redrawing of the tissue architecture is facilitated by the remodeling of ECM by fibroblasts and matrix metalloproteinases (MMPs) and tissue inhibitors of matrix metalloproteinases (TIMPs) [18]. The concept of dysregulation is also applicable to other aspects of wound healing; excessive activity at any phase is deleterious and can be seen clinically as inflammation, uncontrolled proliferation and tumor formation, angiogenesis and vessel growth, fibrotic scarring, or inadequate ECM remodeling.

Stem cell therapies look like they may provide benefits in the healing process after orthopedic injuries due to the regeneration of damaged tissues in Table 2. Some of these stem cells are mesenchymal stem cells (MSCs), and the other is induced pluripotent stem cells (iPSCs). MSCs derived from bone marrow and adipose tissues promote the healing of bone, cartilage, and tendon in fractures, degeneration of cartilage, ligament rupture, and other orthopedic injuries, yet effectiveness shows some variation in several areas and brings potential risks, including immune rejection. iPSCs are stable adult somatic cell derivatives that have the ability, but iPSCs possess certain risks related to the formation of tumors, the high cost of using stem cells, and ethical issues regarding the derivation of stem cells. harDCs are easy to isolate and are abundant in adipose tissue as they promote the repair of tissue damage through direct differentiation and the secretion of growth factors; however, they are characterized by donor-dependent quality and potency. Bone marrow-derived MSCs, although slightly invasive in the process as compared to the other sources are best for bone and cartilage defects in fractures, cartilage injury, and arthritis. Stem cells such as MSCs can also be seeded onto biocompatible scaffolds to support tissue integration and healing in meniscal, cartilage, or other tissue defects, except for the issue of scaffold degradation over time. Every stem cell is characterized by different potential for proliferation, and this aspect must be regulated to have enough stem cells for therapeutic use and, at the same time, to avoid uncontrolled cell growth and formation of tumors. Furthermore, non-invasive MSC populations have been derived to mitigate donor risks of morbidity in other MSCs [19]. Although stem cells are considered a hopeful instrument for improving orthopedic injury healing, several hurdles need resolution for their wider applicability in clinical care.

**Table 2:** Stem Cell Therapies for Orthopedic Injuries outlines key aspects of mesenchymal stem cells (MSCs) and induced pluripotent stem cells (iPSCs), their applications, and associated challenges MSCs: Mesenchymal stem cells; iPSCs: Induced pluripotent stem cells; PRP: Platelet-rich plasma

Stem Cell Type	Source	Action	Applications	Challenges	Proliferation	Harvesting
MSCs	Bone marrow, adipose tissue	Bone, cartilage, tendon repair	Fractures, cartilage, ligaments	Limited results, immune issues	Medium	Invasive/Non-invasive
iPSCs	Reprogrammed somatic cells	Pluripotent, various tissues	Cartilage, tendons, bone repair	Tumors, ethical, costly	High	Non-invasive
Adipose MSCs	Fat tissue	Tissue repair, growth factors	Osteoarthritis, soft tissues	Donor variability	Medium	Non-invasive
Bone Marrow MSCs	Bone marrow aspirate	Bone and cartilage regeneration	Fractures, cartilage, arthritis	Invasive, slow growth	Low	Invasive
MSCs + Scaffolds	MSCs + biocompatible scaffolds	Tissue integration	Meniscus, cartilage defects	Scaffold breakdown	Medium	Depends on the MSC source

Platelet-rich plasma (PRP) and its role in orthopedic healing

Mechanism of PRP Action

Extracellular Matrix (ECM) Effects: PRP has been accepted as a more advanced orthobiologic treatment that applies a high concentration of autologous platelets and related growth factors to the place of the damaged soft tissue and bone [20]. Recent studies also estimate that PRP promotes regenerative healing by specific signaling that targets reinstating ECM and apropos reparative cellular actions [21-23]. The ECM plays a major role in the structural supporting and biochemical modulation of the surrounding cells and is an important site of action of PRP bioactivity.

As mentioned earlier, authors have reported that PRP stimulates ECM synthesis and matters that contain vital bioactive proteins and growth factors [23]. Consequently, the anabolic effects of PRP support the synthesis of proteoglycans, fibronectin, collagen, and other proteins that create the ECM stall [24]. Cell adhesion receptors play a significant role in this structural protein formation since they form biomolecular substrates necessary for cell healing processes, including adhesion, migration, proliferation, and differentiation [25]. However, PRP also inhibits excessive enzymatic degradation of the ECM-reducing structures, frequently in chronic degenerative diseases, such as osteoarthritis [26]. This micro-ecosystem is more amenable to restorative activities via the reinforcement of ECM protein density and PRP integration.

In addition, it has been identified that components of PRP play an important role in boosting cell adhesion matrix interactions that are necessary to transmit a load of biochemical signals, which is essential in the process of tissue healing. Because of its interaction with transmembrane receptors on target cells, both TGF-β and PDGF in PRP stimulate intracellular signal transduction cascades, which lead to gene expression and metabolic processes supporting healing [27]. Thus, signaling between ECM ligands and cell surface receptors contributes to the changes in regenerative cell behaviors. Therefore, the PRPmediated enhancement of cell-matrix interaction enables the orchestrated response required for orthopedic healing.

PRP Action in Osteoarthritis: In the last few years, autologous platelet concentrates, or PRP, have come to prominence in the management of osteoarthritis, mainly because of their anti-inflammatory capabilities and support for chondrogenesis. Thus, the therapeutic work of PRP is delivered through the various growth factors and cytokines that are present in the form of alpha granules of this concentrated platelet [28]. Some of these are transforming growth factor-beta (TGF-β), PDGF, VEGF, insulin-like growth factor (IGF), and interleukin-1 receptor antagonist (IL-1ra), among others [29].

In osteoarthritis, inflammatory biomolecules, for example, tumor necrosis factor-alpha (TNF-α) and interleukin 1β (IL-1β), get over-expressed and destroy the cartilage ECM through the promotion of MMPs, ADAMTS, and other catabolic enzymes [30]. It is proposed that through one or several of the following mechanisms, PRP interacts with these inflammatory signals. First, PRP contains a higher concentration of IL-1ra that competitively occupies IL-1 receptors on chondrocytes, thus blocking IL-1 inflammatory singling pathways [4]. Second, PRP liberates soluble TNF-α receptors, which bind with TNF-α so that it does not get bonded to cell surface receptors [31].

Apart from an anti-inflammatory effect, PRP also promotes the anabolic activity of chondrocytes and the synthesis of the ECM necessary for cartilage health maintenance. Extrinsic signals like TGF-β and IGF bound to the chondrocyte membrane receptors start signal transduction proteins like Smad2/3 that, in turn, trigger the nucleus to transcribe the cartilage matrix [32]. TGF-β also inhibits chondrocyte hypertrophy and terminal differentiation [33]. On top of that, PRP provokes the synthesis of tissue inhibitors of metalloproteinases (TIMPs) that counteract the actions of matrix-degrading metalloproteinases [34]. In the case of osteoarthritis, the proliferative, anti-catabolic, and pro-anabolic effects of signaling molecules released by PRP are worthwhile to maintain the osteoarthritis condition and may also have the potential to stimulate cartilage repair.

**Figure 2:**
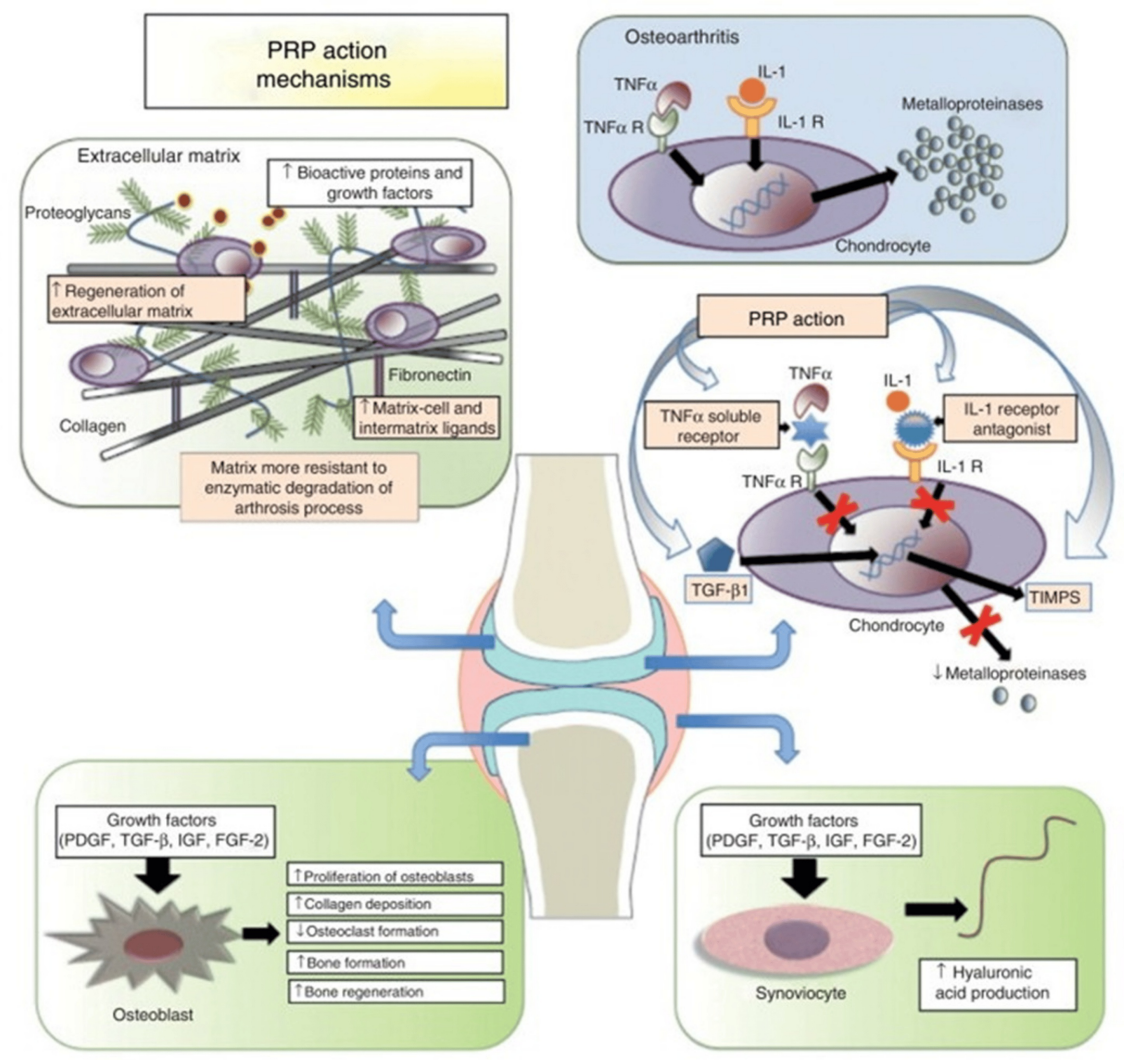
Mechanism of Platelet-Rich Plasma (PRP) action in treating osteoarthritis and its effects on various cells involved in joint health Source: Knop E, Paula L, Fuller R.: Platelet-rich plasma for osteoarthritis treatment. Rev Bras Reumatol Engl Ed. 2015, 56:10.1016/j.rbre.2015.07.002 [47] This article is licensed under a Creative Commons Attribution. PRP: Platelet-rich plasma; TNFα: Tumor necrosis factor-alpha; TNFα R: Tumor necrosis factor-alpha receptor; IL-1: Interleukin-1; IL-1 R: Interleukin-1 receptor; TGF-β1: Transforming growth factor-beta 1; TIMPs: Tissue inhibitors of metalloproteinases; PDGF: Platelet-derived growth factor; IGF: Insulin-like growth factor; FGF-2: Fibroblast growth factor-2

Growth Factors: In recent years, PRP has come into focus as an orthobiologic treatment option for multiple musculoskeletal disorders because of its inherent property to promote the healing of soft tissue as well as bone in Figure 2. The work of PRP is explained by a variety of growth factors and cytokines derived from the α-granules of concentrated platelets, which are released in the activated state [35]. These growth factors affect locally residing progenitor stem cells in a paracrine manner and alter the milieu of tissue regeneration and healing [36].

It is an essential glycophosphoprotein, and for example, PRPs consist of PDGF, transforming growth factor-β (TGF- β), IGF, and fibroblast growth factor-2 (FGF-2) [37]. Of all the growth factors, PDGF and TGF-β are perhaps the most crucial in the processes of tissue repair and regeneration [38]. According to the literature, PDGF acts to pull in stem cells and osteoblasts (bone-developing cells) to the area of injury and supports their growth [39]. TGF-β might help to preserve stability in both ECM and collagen, induce the generation of osteoclasts, and stimulate the growth of new blood vessels and stem cell differentiation into osteoblasts for the regeneration of bone tissue [40]. IGF promotes bone regeneration as it increases collagen synthesis and stimulates osteoblasts population density and differentiation stage [41]. They also found FGF-2 responsible for angiogenic and osteogenic differentiation of stem cells and holds a bigger responsibility for the formation and regeneration of bones [42].

Besides improving the rate of bone healing, PRP has demonstrated a favorable impact on cartilage repair. According to what has been highlighted above concerning the application of PRP, the growth factors stimulate enhanced synthesis of hyaluronic acid by chondrocytes and synoviocytes in joints [43]. Hyaluronic acid is also the main component of synovial fluid and is utilized as a joint lubricant. It is impacted due to its ability to reduce friction between joints and prevent wear and tear of cartilage [44]. For that reason, hyaluronic acid production is improved through PRP to allow for cartilage repair after injuries.

Concisely, through the liberation of several growth factors consisting of osteoinductive, osteogenic as well as chondrogenic forms in the activated platelet, PRP is beneficial as a biological environment for bone and soft tissue regeneration in several orthopedic conditions affecting bone, tendons, ligament, and articular cartilage [45]. PRP therapy has proved to be safe, easy, and convenient, hence making it a possible innovative treatment modality in sports medicine and orthopedics [46].

Growth factors and cytokines

Stem cell-based bone healing and regeneration depend upon several growth factors that are naturally secreted in bodies and control critical cellular functions in motility remodeling [48]. BMPs, for example, BMP-2 and BMP-7, are well known to enhance bone formation actively. BMP-2 is isolated from bone and cartilage tissue and stimulates osteoblast differentiation and new bone formation [49]. It has proved useful in fracture healing and spinal fusion, but the worry is with the likelihood of overstimulation and inflammation [50]. BMP-7 is located in bone and kidney tissue cells and is involved in bone and cartilage formation [51]. Recombinant BMP-7 products have been tested for improving fracture healing in non-union defects and for use in the treatment of arthritis. However, cost factors and limited availability, given the current state of technology, limit their use in the clinic [52].

TGF-β1 and TGF-β2 belonging to The transforming growth factor-beta (TGF-β) family are especially crucial during the process of bone remodeling as well as bone repair [53]. TGF-β1 is accumulated in platelets and bone cells released in response to bone resorption and regulates coupled bone formation [54]. It also modulates soft tissue wound healing but may promote the formation of scar tissue and fibrosis if over-expressed [55]. TGF-β2 is most abundant in cartilage and bone tissue and exerts potent chondrogenic activity as well as promoting cartilage ECM deposition; hence, it may be used for cartilage injury and osteoarthritis treatments [56,57]. Interestingly, all TGF-β isoforms, including TGF-β2, which is expressed by Muller glial cells, may be equally effective in the promotion of fibrosis and scar formation in Table 3.

**Table 3:** The role of growth factors like BMPs and TGF-β in bone healing BMP: Bone morphogenetic protein; TGF-β: Transforming growth factor-beta; VEGF: Vascular endothelial growth factor; IGF-1: Insulin-like growth factor-1; PDGF: Platelet-derived growth factor

Growth Factor	Source	Function	Target Tissue	Clinical Applications	Challenges
BMP-2	Bone, cartilage	Stimulates bone formation	Bone	Fracture healing, spinal fusion	Overstimulation, inflammation
BMP-7	Bone, kidneys	Bone and cartilage repair	Bone, cartilage	Non-union fractures, arthritis	Costly, limited availability
TGF-β1	Platelets, bone cells	Regulates bone remodeling	Bone, soft tissues	Fracture repair, soft tissue healing	Scar formation, fibrosis
TGF-β2	Bone, cartilage	Stimulates cartilage formation	Cartilage, bone	Cartilage repair, joint injuries	Excessive scarring
VEGF	Endothelial cells	Promotes blood vessel growth	Bone, soft tissues	Fracture healing, tissue repair	Uncontrolled growth, tumors
IGF-1	Liver, bone cells	Stimulates bone growth	Bone, muscle	Bone regeneration, muscle repair	Short half-life, side effects
PDGF	Platelets, bone cells	Cell proliferation and healing	Bone, soft tissues	Fracture healing, soft tissue repair	Inflammation, overproduction

VEGF, IGF-1, and PDGF, though, have growth actions that are complementary to erythropoietin in the process of bone healing. VEGF from endothelial cells promotes angiogenesis to ensure blood flow during fracture repair [58]. However, high and or low levels of VEGF have implications for the creation of unwanted blood vessels, which can contribute to the formation of tumors [59]. The hepatocyte and skeletal osteokine IGF-1 promotes bone formation and mineralization by acting directly on the osseous tissue formed by the liver and osteoblast [60]. However, its clinical application is somewhat hampered by a short half-life and possible undesirable effects [61]. PDGF present in platelets and bone cells discharged following such injuries directs tissue repair processes such as cell division [62].

Gene therapy approaches for orthopedic repair

**Figure 3:**
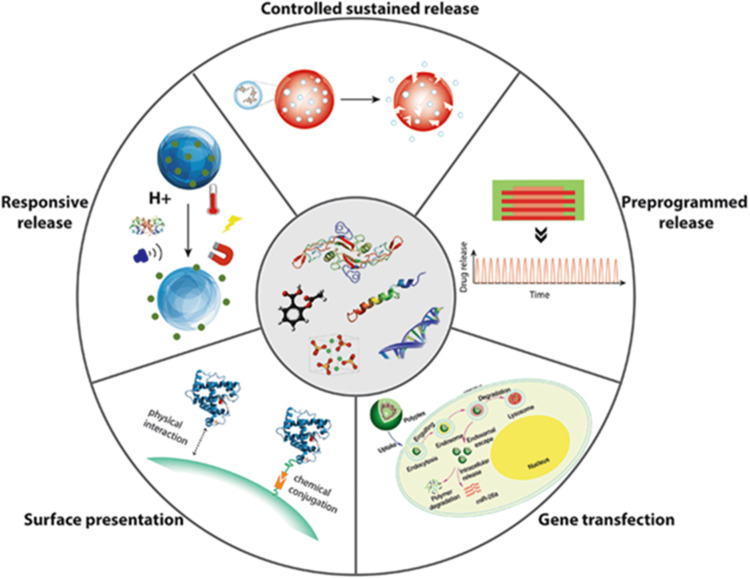
Various drug delivery mechanisms in biomedical applications Source: Dang M, Saunders L, Niu X: Biomimetic delivery of signals for bone tissue engineering. Bone Res. 2018, 6:25. 10.1038/s41413-018-0025-8 [72] This article is licensed under a Creative Commons Attribution

Some of the mechanisms include feedback systems that allow the release of the therapeutic agents at designed rates or according to superimposed commands from other sections of the body in Figure 3. Such novel systems have been developed to optimize the treatment results, optimize the safety measures, and establish individualized treatments [63]. One of them is the controlled, sustained release, where drugs are coated in materials that will allow the drug to disintegrate slowly over an extended period [64]. This mechanism also holds a steady balance in miscibility and biological availability and helps to prevent toxicity due to excessive and sudden dose accumulation. They release the drugs in cyclic patterns with standpoints between drug concentrations maintained programmatically to elicit the required characteristics of drug delivery [65]. This pulsatile profile replicates circadian rhythms and biological responses that help the drugs exert their therapeutic effects [66]. Gene transfection is based on introducing DNA or RNA into target cells utilizing vectors [67]. However, once internalized, the nucleic acids can deliver therapeutic proteins or change disease genes [68]. Surface functionalization exposes drug molecules to biological media on surfaces of materials where they physicochemically adhere [69]. This approach enables localized delivery and sensor biointerfaces [70]. Last, controlled release systems release drugs in response to external changes in pH, temperature, light, or any other stimuli [71]. This “smart” delivery enhances targeting and controls consistent with the physiological reaction [71]. The above-advanced mechanisms add value to pharmacotherapy, keeping patients comfortable through various techniques that enable sustained pulsatile targeted or responsive drug release profiles. It also permits the delivery of biologics such as genes and cells, which increases the distinct therapeutic choices available to the public.

Role of extracellular vesicles (EVs) in regenerative orthopedic therapies

An understanding of the growth factors is that they are useful for promoting the bone regeneration processes in osteotomies and speeding up the rates of healing in diverse fields, but some obstacles must similarly be encountered before these potential applications become mainline solutions consistently in Table 4. The Bone morphogenetic proteins (BMPs) stimulate osteoblast differentiation and bone formation. BMP-2 targets bone regeneration through osteoblast differentiation with an emphasis on a controlled delivery system for fracture and spinal fusion [72,73]. Potential complications include inflammation or over-activation of bone formation by BMP-2 [74]. BMP-7 is involved in cartilage repair through chondrogenesis, and more so, clinical trials have been made to test it in osteoarthritis, non-union fractures, and cartilage injury and damage. TGF-β1 and TGF-β2 are involved in the BM process, being responsible for cell proliferation and differentiation. Despite providing additional cures in bone tissue remodeling, TGF- β1 has the potential for fibrosis and scarring, though research on the new scaffold for its delivery in bone, as well as soft tissue repair, is ongoing [75, 76] TGF-β2 has been proven to effectively induce cartilage repair through regulating ECM synthesis with current researches aimed at reducing scar tissue formation for joint repair and cartilage injury treatment [77]. However, systemic application of TGF-β2 poses certain concerns of excessive scar formation.

**Table 4:** Emerging Research on Their Therapeutic Potential, focusing on the therapeutic potential of growth factors in bone healing and regenerative medicine ECM: Extracellular matrix; VEGF: Vascular endothelial growth factor; TGF-β: Transforming growth factor-beta; IGF-1: Insulin-like growth factor-1; PDGF: Platelet-derived growth factor; BMP: Bone morphogenetic protein

Growth Factor	Therapeutic Potential	Mechanism	Current Research Focus	Applications	Challenges
BMP-2	Enhances bone regeneration	Induces osteoblast differentiation	Research on controlled delivery systems	Fracture healing, spinal fusion	Risk of overstimulation, inflammation
BMP-7	Promotes cartilage repair	Stimulates chondrogenesis	Ongoing clinical trials for osteoarthritis	Non-union fractures, cartilage repair	High cost, limited use in clinics
TGF-β1	Improves bone remodeling	Regulates cell proliferation and differentiation	Novel scaffolds for targeted delivery	Bone and soft tissue repair	Potential for fibrosis and scarring
TGF-β2	Stimulates cartilage regeneration	Modulates ECM production	Research on minimizing scar formation	Joint repair, cartilage injuries	Excessive scar tissue development
VEGF	Supports vascularization	Promotes angiogenesis	Focus on combination therapies with scaffolds	Fracture healing, tissue repair	Uncontrolled angiogenesis, tumor risk
IGF-1	Enhances bone growth	Activates osteoblasts and muscle cells	Studies on improving delivery methods	Bone regeneration, muscle repair	Short half-life, systemic effects
PDGF	Accelerates wound healing	Stimulates cell proliferation and ECM production	Research on topical applications	Fracture healing, soft tissue repair	Risk of excessive inflammation

VEGF is therefore affiliated with vascularization and stimulates angiogenesis. Fracture healing and tissue repair requiring the formation of new blood vessels have been explored utilizing combination delivery systems for scaffolds [78, 79]. However, controlling the extent of VEGF-induced angiogenesis remains problematic, as well as the risks of tumorgenicity. IGF-1 stimulates bone formation and muscle cell proliferation by activation of osteoblasts and muscle cells, respectively. This review argues that enhancing the delivery system of IGF-1 could help it be used for bone regeneration and muscular repair [80]. However, IGF-1 has a short half-life and has other non-local effects. Finally, PDGF has a role in wound healing as it helps in cell division and deposit of ECM machinery. Other targeted topical uses of PDGF are under investigation for fracture healing and soft tissue injury repair [81]. However, controlling its activity to moderate inflammation proves challenging. Therefore, despite the therapeutic potential of a few growth factors, issues including higher costs, poor effectiveness, transportation, unwanted proliferation, uncontrollable outcomes, neofibrosis, contraction, and inflammatory responses currently hinder their use and enhance the idea of regenerative medicine.

Challenges and future directions

Regenerative medicine is a relatively young science that studies the process of restoring the functions of organ and tissue injuries. The application of regenerative medicine for the treatment of different types of sports-related orthopedic injuries involving bone, cartilage, tendon, and ligament has been rising [82]. Nonetheless, there are several potential problems and drawbacks linked with existing regenerative treatments that have to be discussed and further investigated.

Challenges

Limited Cell Sources: An important issue is a restricted number of cell sources for use in regenerating strategies. Sources of autologous cells, for instance, bone marrow and adipose tissue, are scarce, and their harvest results in donor-site morbidity [83]. However, pluripotent or multipotent stem cell lines have problems with histocompatibility, tumorigenicity, and their ability to maintain differentiation potential in several passages [84]. Further studies are required to specify native unaltered autologous cell sources, which may produce numerous orthopedic tissue types.

Lack of Regulation Systems: Mostly, there are inadequate elucidations of molecular and cell biology that govern organized tissue formation and particularly tissue remodeling after any kind of injury [85]. Failure to demystify these regulatory programs makes it difficult to properly design cell- and biomaterial-based structures to switch on tissue regeneration once implanted in vivo appropriately.

Short-Lasting Benefits: It was found out that most of the current regeneration theories such as stem cells, PRP and scaffolds have been found to act as symptomatic with verifiable improvements observed that seem to decline after 1-2 years of the treatment. The long-term benefits of regenerative therapies for orthopedic injuries remain challenging to realize [86].

High Costs and Regulatory Barriers: The effort of translating novel regenerative therapies, such as cell and gene therapies, to patients has been associated with high costs and slow regulatory processes [87]. Many of the most promising treatment methods are still at best considered investigational or are available only in limited clinical trials. More efficient and innovative models of financing and approval procedures should be considered to speed up translation.

Future directions

Biomaterials and Scaffolds

New possibilities in the field of biomaterials allow designing bioresponsive structures that can stimulate tissue self-healing [88]. Scaffolds could be tailored to release drug molecules of interest or designed to deliver physical and chemical signals that would attract host cells and organize the processes of repair and tissue regeneration.

3D Bioprinting

Tissue engineering methods enable the forming of complex structures of cells and matrix components that can represent the structural organization of the specific tissue type [89]. As bioprinting technology improves, appropriate bioinks and patient-specific tissue grafts could be produced with a potential for implantation for focal injuries or defects.

In-situ Regeneration

The encouraging technique to induce regeneration in situ is by reprogramming resident cells or altering host response appears to be a less invasive approach compared to cell transplantation [90]. Strategies designed to modulate epigenetic environment and mechanical and chemical signals that convert inflammation to regeneration should be explored more in the context of multiple orthopedic injuries.

Gene and RNA Therapies

Autologous stem cell release, gene-modified cell therapies, and the direct delivery of small regulatory RNA molecules can be potentially shown to modify cell force and activity in the context of regeneration [91-93]. There should be attempts to develop safer means for gene delivery and identify the right master regulator genes to target.

The current regenerative therapies are effectively therapeutic, but future enhancement in cell sources, mechanisms, construct, and translation methodology are areas that require much attention to make regenerative medicine a standard of care for different orthopedic debilitating injuries. Further research and development in this area of study that links the principles of bioengineering, stem cell engineering, materials science gene therapy, and tissue repair all into one are essential for the production of more effective and translatable ideas and inventions.

## Conclusions

Regenerative medicine offers promising advancements in orthopedic injury management through stem cells, PRP, growth factors, and gene therapies. These approaches enhance tissue healing and repair, yet challenges like standardization, long-term efficacy, and regulatory hurdles remain. Continued research and technological innovations, including biomaterials and gene editing, are essential for translating these therapies into mainstream clinical practice. With interdisciplinary collaboration, regenerative medicine has the potential to revolutionize orthopedic treatment and improve patient outcomes.
